# Waste Glass Powder as a Circular-Economy Precursor in Geopolymer Binders

**DOI:** 10.3390/ma19071357

**Published:** 2026-03-29

**Authors:** Sri Ganesh Kumar Mohan Kumar, John M. Kinuthia, Jonathan Oti, Blessing O. Adeleke

**Affiliations:** Faculty of Computing, Engineering and Science, University of South Wales, Pontypridd CF37 1DL, UK

**Keywords:** waste glass powder, alkali-activated materials, geopolymer binders, reaction–transport coupling, gel chemistry, durability, pore structure, alkali–silica reaction, ageing, circular economy

## Abstract

The transition toward low-carbon and resource-efficient construction materials has intensified interest in geopolymer binders incorporating industrial and post-consumer wastes. Waste glass powder (WGP), a silica-rich component of the global glass waste stream, has emerged as a promising circular-economy precursor in alkali-activated systems; however, reported durability trends remain inconsistent and are often interpreted without mechanistic integration. This review synthesises current knowledge of WGP reactivity, gel chemistry, and long-term performance through an explicit reaction–transport–ageing (R–T–A) framework that links dissolution behaviour and phase assemblage development to pore connectivity, ion ingress, and time-dependent degradation. Under alkaline activation, the amorphous structure of WGP promotes silica release, modifying Si/Al ratios and governing the formation of N-A-S-H or hybrid N-A-S-H/C-(A)-S-H gels. These reaction products determine transport characteristics and ageing evolution, which collectively control chemical resistance, chloride ingress, alkali–silica reaction-type instability, and dimensional stability. Variability across studies is shown to arise from imbalances in particle fineness, replacement level, precursor chemistry, and activator design rather than intrinsic inconsistency in WGP behaviour. The R–T–A framework clarifies how reaction completeness, pore network architecture, and long-term phase stability interact to produce system-dependent durability outcomes. WGP demonstrates strong potential as a circular-economy precursor in alkali-activated binders; however, reliable structural application requires durability-informed mix design grounded in coupled reaction–transport–ageing mechanisms and supported by extended exposure testing under realistic service conditions.

## 1. Introduction

Alkali-activated materials (AAMs), including geopolymer binders, are increasingly investigated as alternatives to ordinary Portland cement (OPC) due to their potential for reduced greenhouse-gas emissions and their ability to incorporate industrial and post-consumer waste streams [1]. The performance of these binders is governed primarily by precursor chemistry, activator composition, and curing regime, which together control dissolution behaviour, gel formation, pore structure development, and long-term durability [1,2,3]. In this review, the term *geopolymer* is used in a broad sense to describe alkali-activated aluminosilicate binders derived from waste-based precursors.

As the availability of conventional aluminosilicate precursors such as fly ash and slag becomes increasingly constrained by regional supply variability, attention has shifted toward alternative silica-rich resources [2,3,4]. Waste glass powder (WGP) represents one such candidate. Waste glass is an abundant but underutilised stream, with significant quantities remaining unrecycled due to contamination, colour variability, and inefficiencies in collection and processing systems [5,6,7,8]. When processed into a fine powder, WGP consists predominantly of amorphous silica and can dissolve under alkaline conditions, contributing to pozzolanic reactions in cementitious systems and to binder formation in alkali-activated matrices [2,4,9,10]. Broader durability challenges in conventional concrete systems, including damage evolution under freeze–thaw and salt erosion environments [11], alongside efforts to enhance toughness through improved fibre–matrix interface bonding in ultra-high-performance concrete [12], underscore the importance of microstructural stability in achieving reliable long-term durability of cementitious materials.

Despite this potential, WGP differs fundamentally from conventional geopolymer precursors. It typically exhibits high SiO_2_ content with low intrinsic Al_2_O_3_ and CaO concentrations, which strongly influence dissolution kinetics, gel chemistry, Si/Al ratios, and microstructural development during activation [2,3,9,13]. As a result, WGP alone often struggles to form a sufficiently cross-linked geopolymeric network because of its limited alumina availability and low calcium contribution. In practice, WGP is therefore commonly incorporated in blended systems, particularly with calcium-rich precursors such as ground granulated blast furnace slag (GGBS), to enhance early reaction kinetics and promote structural stability [5,14,15,16]. In such blended systems, mixed gel phases including N-A-S-H and hybrid (N, C)-A-S-H networks can develop depending on activator chemistry, precursor proportions, and curing conditions [17,18,19,20].

Although several studies report potential benefits associated with WGP incorporation, including microstructural refinement, modified pore structure, and in some cases improved mechanical or durability indicators, reported outcomes remain highly inconsistent [5,19,21,22,23]. Strength development, transport behaviour, shrinkage, and chemical stability are all sensitive to particle fineness, replacement level, precursor balance, and alkali dosage, leading to contradictory findings across studies [2,3,24,25,26]. Conflicting observations regarding alkali–silica reaction (ASR) susceptibility and expansion in both OPC-based and alkali-activated systems incorporating glass further suggest that silica dissolution and secondary gel formation under high-alkali conditions are not yet fully resolved [4,7,10,15,27].

Durability assessment represents a particular weakness in the existing body of work on WGP-based alkali-activated binders. Many investigations focus on a narrow set of indicators such as water absorption, acid or sulphate resistance, or isolated transport tests, and frequently use paste or mortar specimens rather than concrete [4,14,17,22,24,27]. Comprehensive evaluations of transport-related durability (e.g., sorptivity, permeability, chloride diffusion, electrical resistivity), dimensional stability (drying shrinkage, expansion), and microstructure–durability relationships remain relatively scarce, particularly for high WGP contents and WGP–GGBS blended systems [3,5,14,20,28]. Long-term ageing, coupled deterioration mechanisms (e.g., combined ion ingress and shrinkage), leaching behaviour, and performance under realistic exposure conditions remain only partially explored [4,29,30].

Given the fragmented and sometimes contradictory nature of existing findings, a mechanistically grounded synthesis is required. This review critically evaluates WGP-based alkali-activated binder systems through the coupled lenses of dissolution and gel evolution, pore structure development, and durability-governing transport and ageing processes, with particular emphasis on WGP–GGBS blends where practical implementation is most viable. The review clarifies dissolution behaviour and gel formation pathways; evaluates chemical and physical durability across exposure conditions; and identifies research gaps that must be addressed to enable reliable structural application of geopolymer concretes incorporating waste glass. In calcium-rich systems discussed here, the term geopolymer is used to denote alkali-activated binders exhibiting hybrid reaction products rather than purely aluminosilicate networks.

### 1.1. Distinct Contribution of This Review

Unlike previous reviews that primarily summarise mechanical performance trends, recycling pathways, and isolated durability indicators of waste glass powder (WGP), the present work develops a mechanistic synthesis centred on durability evolution. Earlier WGP reviews typically treat strength, transport properties, and chemical resistance as independent performance metrics, with limited integration of reaction chemistry, pore structure development, and long-term ageing mechanisms.

The primary conceptual contribution of this review is the introduction and application of a reaction–transport–ageing (R–T–A) framework that links precursor dissolution, gel chemistry, pore network architecture, and degradation processes within a unified interpretative structure. This framework enables apparently inconsistent durability outcomes reported in the literature to be understood as system-dependent consequences of coupled physicochemical mechanisms rather than as contradictory experimental observations.

This review is also distinct from the authors’ recent work on geopolymer chemistry [1]. The earlier article focused on fundamental geopolymerization mechanisms, precursor chemistry, and reaction pathways across a broad range of aluminosilicate systems. It did not specifically examine WGP as a system-dependent precursor, nor did it address durability evolution governed by transport processes and long-term ageing phenomena. The present work, therefore, extends beyond reaction chemistry to evaluate how WGP modifies microstructure, transport behaviour, and environmental degradation resistance.

Furthermore, this review differentiates low-calcium aluminosilicate binders from calcium-rich hybrid alkali-activated systems, clarifying how WGP influences gel assemblage, pore connectivity, and degradation susceptibility differently across these chemistries. By integrating reaction chemistry, transport physics, and ageing mechanisms, the article provides a durability-informed interpretative framework that has not previously been synthesised in WGP literature.

Accordingly, the objective of this review is not to provide an encyclopaedic survey of all WGP studies, but to deliver a mechanistically grounded synthesis that reconciles durability variability and supports reliability-oriented application of WGP in alkali-activated binder systems.

### 1.2. Terminology and Classification Framework

Terminology in alkali-activated binder research is not uniformly standardised, particularly regarding the use of the term “geopolymer,” which is often applied inconsistently across chemistry-focused and engineering-focused literature [31]. In strict chemical usage, geopolymers refer to low-calcium alkali-activated aluminosilicate binders whose reaction products are dominated by sodium aluminosilicate hydrate (N-A-S-H) gel networks formed through polycondensation of aluminosilicate species. These materials are characterised by highly cross-linked three-dimensional frameworks with minimal calcium participation in the binding phase [32].

By contrast, the broader category of alkali-activated materials (AAMs) includes binders derived from a wide range of aluminosilicate and calcium-rich precursors activated by alkaline solutions. Depending on precursor chemistry, AAMs may form sodium aluminosilicate hydrate (N-A-S-H), calcium–aluminosilicate hydrate (C-(A)-S-H), or hybrid gel assemblages containing both reaction products [33].

Hybrid alkali-activated binders, particularly those incorporating ground granulated blast furnace slag (GGBS) or high-calcium fly ash, commonly develop coexisting N-A-S-H and C-(A)-S-H type gels. These systems differ chemically and microstructurally from low calcium geopolymers because calcium modifies network polymerisation, reaction kinetics, and long-term phase stability [34].

The literature often uses “geopolymer” as an umbrella term encompassing all alkali-activated binders regardless of calcium content, especially in engineering and applied studies. To avoid ambiguity while maintaining continuity with established usage, the present review adopts the following classification framework, summarised in Table 1.

- **Geopolymers** refer specifically to low-calcium alkali-activated aluminosilicate binders dominated by N-A-S-H gel networks [32].
- **Alkali-activated materials (AAMs)** denote the broader class of binders formed through alkaline activation of aluminosilicate or calcium-rich precursors [33].
- **Hybrid alkali-activated binders** describe calcium-containing systems that develop mixed N-A-S-H/C-(A)-S-H gel assemblages [34].

Terminology inconsistencies in the literature and the broad engineering use of the term “geopolymer” have been widely discussed [31].

## 2. Research Landscape and Thematic Fragmentation in Waste Glass Powder Studies

**Figure 1:**
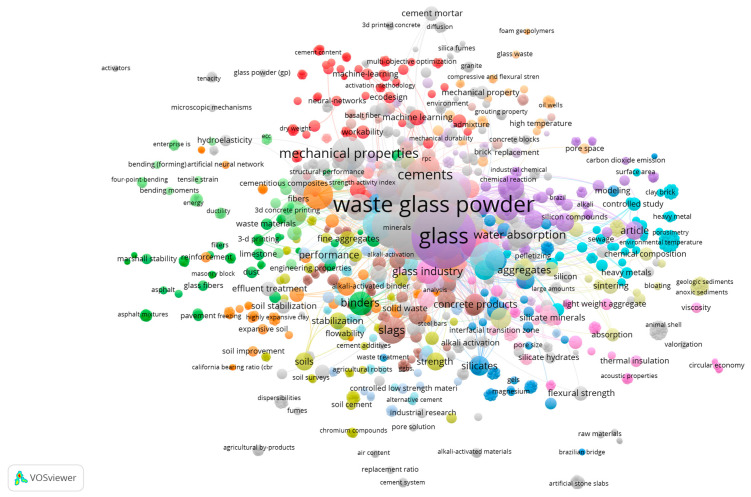
Keyword co-occurrence network of publications related to waste glass powder, showing the main research themes and clusters generated using VOS viewer version 1.6.20.

Research on waste glass powder (WGP) has expanded rapidly over the past decade; however, this growth has occurred along multiple partially disconnected trajectories rather than within a unified mechanistic framework. Bibliometric mapping of the published literature (Figure 1) demonstrates that WGP research is distributed across distinct thematic clusters corresponding to: (i) geopolymer and alkali-activated binders; (ii) mechanical and durability performance of cementitious concretes; (iii) soil stabilisation and geotechnical applications; (iv) environmental remediation and waste-treatment uses; and (v) recycling and circular-economy processes.

The largest cluster is centred on cementitious performance indicators such as compressive strength, water absorption, and mechanical properties. A separate but overlapping cluster captures geopolymer and alkali-activated systems, where emphasis shifts toward activator chemistry, gel formation, and phase evolution. Additional clusters focus on soil improvement and environmental engineering applications, often prioritising stabilisation performance or contaminant immobilisation rather than binder chemistry. Circular-economy and waste-management studies form another grouping, typically evaluating diversion rates, recycling efficiency, and sustainability metrics.

This thematic dispersion is not merely descriptive; it has direct consequences for how WGP performance is interpreted. Different research communities adopt different specimen scales, curing regimes, precursor systems, and evaluation criteria. Sustainability-driven studies frequently emphasise embodied carbon or waste diversion, while materials-focused investigations prioritise early-age strength or isolated durability indicators. Geotechnical applications rely on distinct testing protocols and performance benchmarks compared with structural concrete research. As a result, mechanistic consistency across studies is limited, and direct comparison of durability outcomes is often invalidated by differences in system chemistry, activator design, curing conditions, and test methodology.

**Figure 2:**
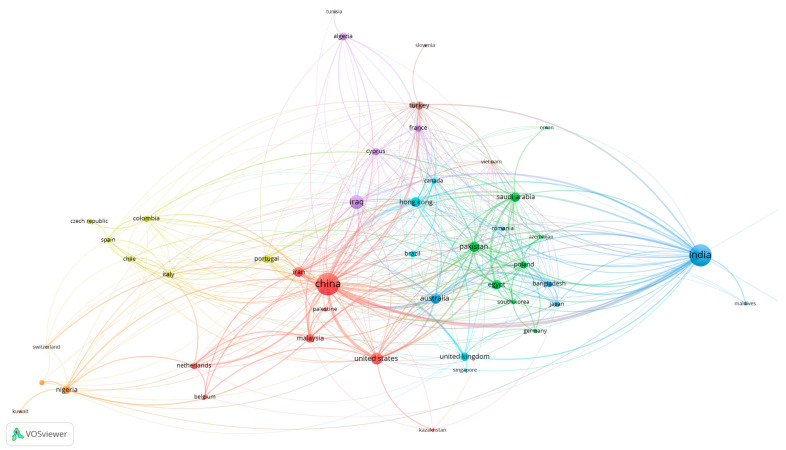
Country-level co-authorship collaboration network showing global research partnerships in waste glass powder studies using VOS viewer version 1.6.20.

At the international level, collaboration networks (Figure 2) further illustrate structural fragmentation. China functions as a central hub in experimental research on WGP in cementitious and alkali-activated systems, while countries such as India exhibit high publication volumes but more regionally concentrated collaboration patterns. European research networks demonstrate stronger inter-country integration but frequently focus on recycling performance and circular-economy frameworks. These geographically clustered research ecosystems tend to employ locally available precursors and region-specific practices, reinforcing variability in mix design, activator chemistry, and reporting standards.

The dispersed and heterogeneous structure of WGP research provides a structural explanation for the contradictory conclusions reported in the literature regarding reactivity, transport behaviour, shrinkage, alkali–silica reaction risk, and long-term durability. Apparent inconsistencies are frequently not intrinsic to WGP itself, but instead arise from differences in precursor balance, aluminium availability, calcium content, activator modulus, specimen scale, and exposure regime. Without mechanistic alignment, performance metrics are compared across fundamentally different chemical systems.

This fragmentation underscores the need for synthesis approaches that prioritise reaction mechanisms, gel chemistry, and transport processes over isolated strength or durability indicators. A mechanistically integrated framework is required to reconcile system-dependent variability and to interpret durability evolution across diverse WGP-based binders. The reaction–transport–ageing (R–T–A) framework adopted in the following sections is proposed specifically to address this structural fragmentation by linking dissolution behaviour, phase assemblage development, pore connectivity, and long-term degradation into a unified interpretative model.

## 3. Global Context of Waste Glass Generation, Recycling, and Valorization

Glass is widely used in packaging, construction, and industrial applications due to its chemical durability, impermeability, and mechanical performance [35]. Global glass consumption has increased steadily with population growth and urbanisation; however, despite glass being theoretically recyclable without material degradation, a substantial fraction of post-consumer glass is not recovered through closed-loop recycling systems [21]. Contamination, colour mixing, compositional variability, and limitations in collection and sorting infrastructure frequently prevent reintegration of recovered glass into primary glass manufacturing streams [6,7].

As a result, large volumes of waste glass are diverted to landfills or low-value applications, particularly in cases involving mixed-colour or contaminated cullet. Even in regions with relatively high collection efficiencies, such glass streams often fail to meet quality requirements for remelting and are excluded from closed-loop recycling [36]. In many developing economies, these challenges are compounded by fragmented collection systems and limited processing capacity, leading to persistently under-recovered glass waste streams [6,7].

From a construction materials perspective, these systemic limitations have two important implications. First, waste glass available for secondary applications is inherently heterogeneous in composition, colour, and impurity content. Second, this heterogeneity is structural rather than incidental, meaning that large-scale valorisation strategies must accommodate variability rather than rely on narrowly specified feedstocks. Consequently, alternative utilisation pathways that tolerate compositional diversity are required if waste glass is to be diverted from landfill in meaningful quantities.

Grinding waste glass into finely divided powder provides one such pathway, enabling its use as a silica-rich component in cementitious and alkali-activated binder systems. Unlike remelting, powder-based utilisation is less sensitive to colour and compositional variation, while allowing amorphous silica to participate in pozzolanic or alkali-activation reactions [2,3,4]. This route, therefore, aligns circular-economy objectives with materials engineering requirements while directly influencing reaction chemistry, gel development, and durability performance in geopolymer binders. Table 2 summarises representative country-level glass packaging recovery data to illustrate the scale of under-recovered glass streams and the persistence of heterogeneous waste glass availability across regions.

## 4. Characteristics of Waste Glass Powder (WGP)

### 4.1. Origin and Processing of Waste Glass

Waste glass powder (WGP) is produced by crushing, grinding, and milling post-consumer or industrial glass cullet into a fine amorphous powder. Reduction in particle size increases the specific surface area and enhances dissolution under alkaline conditions, enabling WGP to function as a reactive silica source in cementitious and alkali-activated binder systems [3,7,9,36]. Mixed-colour, contaminated, or compositionally inconsistent glass streams that are unsuitable for remelting are commonly utilised for WGP production, providing a practical valorization route for low-value glass waste [7,36].

#### Types of Waste Glass Relevant to Geopolymer Systems

Commercial waste glass streams are dominated by soda–lime silicate glass, which accounts for the majority of container and flat glass products worldwide [6,7]. Other glass types, including borosilicate and aluminosilicate glasses, are present in smaller quantities and are more commonly associated with specialised applications such as laboratory ware, lighting, and technical products [6]. These glass families differ in network structure, modifier content, and chemical durability, leading to distinct dissolution behaviour under alkaline activation [3,6,45]. Consequently, the composition of the parent glass represents an important controlling factor in the reactivity of waste glass powder, and distinctions between glass types provide essential context for interpreting subsequent discussions on dissolution kinetics, gel formation, and durability performance.

### 4.2. Physical Properties of Waste Glass Powder

The physical characteristics of waste glass powders reported in the literature are summarised in Table 3, which compiles experimentally measured properties including density, particle size distribution, and specific surface area for glass powders used in cementitious and alkali-activated systems [9,13,17,27,46,47,48,49]. These parameters play a critical role in controlling the reactivity of waste glass powders, influencing both the dissolution behaviour of silica in alkaline environments and the packing characteristics of blended binder systems.

Across the studies reviewed, the true density of waste glass powders generally falls within a relatively narrow range of approximately 2.46–2.53 g/cm^3^, which is consistent with the typical density of soda-lime glass widely used in container production [13,46,47,49]. The similarity in density values across different recycling streams indicates that grinding processes and particle size reduction have limited influence on the intrinsic material density of glass powders. An exception is observed in the silica-rich glass powder reported by Kameche et al. [48], which exhibited a higher density of 2.97 g/cm^3^, likely reflecting differences in chemical composition associated with silica-rich glass formulations.

In contrast to density, the particle size of waste glass powders varies substantially between studies, largely depending on grinding intensity and the intended application of the material. The median particle size (D50) reported in Table 3 ranges from approximately 4.65 µm to 34.78 µm, indicating that glass powders can be produced over a wide fineness spectrum. Very fine powders were obtained through intensive milling processes, as demonstrated for fluorescent lamp glass powder used in geopolymer systems [17] and further-milled powders developed for high-performance concrete applications [49]. Conversely, several studies report moderately ground glass powders with D50 values between 15 and 25 µm, which appear to represent a practical balance between grinding energy requirements and sufficient fineness to enhance pozzolanic reactivity [46,47].

A clear inverse relationship can also be observed between particle size and specific surface area, with finer powders exhibiting substantially higher Blaine fineness values. For example, the fluorescent lamp glass powder with a median particle size of 4.65 µm exhibited a Blaine surface area exceeding 1000 m^2^/kg [17], whereas coarser powders with particle sizes greater than 30 µm typically exhibit surface areas below 200 m^2^/kg, as reported for industrially milled glass powders used in high-performance concrete [49]. Similar trends are observed for size-fractionated powders investigated by Mirzahosseini and Riding [9], where decreasing particle size results in progressively higher Blaine fineness values.

Overall, the compiled data indicate that most studies utilise waste glass powders with median particle sizes between approximately 10–25 µm and Blaine fineness values in the range of 250–600 m^2^/kg, suggesting that these ranges provide an effective compromise between grinding energy consumption and the surface area required to promote dissolution and reaction of amorphous silica. Consequently, while the intrinsic density of glass powders remains relatively constant, particle size distribution and surface area emerge as the primary physical parameters controlling the reactivity and performance of waste glass powders in cementitious and alkali-activated systems.

### 4.3. Chemical Composition of Waste Glass Powder

The chemical composition of waste glass powder (WGP) is dominated by amorphous SiO_2_, with alkali and alkaline-earth oxides such as Na_2_O and CaO present as network modifiers, alongside minor quantities of Al_2_O_3_, MgO, and K_2_O depending on the parent glass and degree of contamination. A comparative summary of reported oxide compositions is presented in Table 4, illustrating the substantial compositional variability of WGP sources used in geopolymer and alkali-activated binder systems.

Across the literature, SiO_2_ contents commonly range from approximately 68 wt.% to above 85 wt.% (Table 4), reflecting differences in glass type and processing history [13,17,22,24]. This high silica content underpins the suitability of WGP as a supplementary silica source in alkali-activated systems. However, the consistently low Al_2_O_3_ contents, typically below 3 wt.%, confirm that WGP alone cannot sustain geopolymer network formation and must be combined with alumina-rich precursors such as fly ash, slag, or metakaolin to enable stable N-A-S-H or hybrid gel development [32,45].

Alkali oxides, primarily Na_2_O, are present at appreciable levels in many WGP sources, with reported values ranging from approximately 7 to nearly 20 wt.% (Table 4). These alkalis enhance glass dissolution under alkaline activation and can accelerate early reaction kinetics. However, elevated alkali contents also influence pore solution chemistry and may increase the risk of alkali mobility, efflorescence, or long-term chemical instability [53] if not balanced by sufficient aluminium incorporation into reaction products [4,27]. This dual role of alkalis helps explain the variability in reported durability and serviceability outcomes for WGP-containing geopolymer systems.

Calcium content represents a further source of variability with significant implications for reaction pathways. While WGP is commonly considered a low-calcium material, CaO contents in the range of approximately 7–12 wt.% are frequently reported (Table 4), particularly for soda–lime glass. In blended systems, this calcium can contribute to the formation of calcium–aluminosilicate hydrate or hybrid N-A-S-H/C-(A)-S-H gel assemblages when combined with slag or high-calcium fly ash, altering pore structure development and durability behaviour [14,18,20]. Excessive calcium availability, however, may increase susceptibility to decalcification under aggressive chemical exposure [32,45].

The compositional variability evident in Table 4 underscores a critical limitation in the current literature: WGP is often treated as a chemically uniform material despite substantial differences in oxide composition, alkali content, and loss on ignition. These differences directly affect dissolution behaviour, gel chemistry, and long-term durability, and they provide a mechanistic explanation for conflicting performance trends reported across studies. Meaningful comparison of WGP-based geopolymer systems, therefore, requires explicit chemical characterisation and careful integration of WGP with complementary precursors rather than reliance on nominal replacement levels alone.

### 4.4. Reactivity and Reaction Mechanisms of WGP in Geopolymer Systems

The reactivity of waste glass powder (WGP) in geopolymer systems differs fundamentally from that of conventional aluminosilicate precursors such as fly ash or metakaolin [45], owing to its silica-rich but aluminium-deficient composition (Table 4) [6,45]. As a result, WGP does not act as a complete geopolymer precursor but instead influences reaction processes through its dissolution behaviour and subsequent interaction with alumina-bearing materials [1,2,45]. Understanding the mechanisms governing WGP reactivity is therefore essential for interpreting its role in gel formation, microstructural development, and the downstream durability performance of WGP-based geopolymer binders.

This section focuses on the key reaction mechanisms controlling WGP behaviour under alkaline activation, with particular emphasis on dissolution kinetics, the influence of particle fineness and glass chemistry, and the role of activator composition. These mechanisms determine the availability of silicate species in the pore solution and govern how effectively WGP-derived silica is incorporated into geopolymer or hybrid gel networks [45]. Among these processes, alkaline dissolution of the glass phase represents the critical rate-controlling step [3,9,45] and is examined in detail in the following subsection. In addition to participating in early dissolution reactions, a portion of WGP particles may remain partially unreacted within the hardened matrix [3,9,45]. These residual particles can contribute to microstructural densification through a filler effect and may also act as a delayed silica source during later stages of reaction as dissolution progresses under alkaline conditions [9,17,45].

### 4.5. Dissolution Behaviour of Waste Glass Powder Under Alkaline Activation

The reactivity of waste glass powder (WGP) in geopolymer systems is governed primarily by its dissolution behaviour under highly alkaline conditions. Waste glass consists predominantly of an amorphous silicate network in which Si–O–Si bonds are partially disrupted by network-modifying oxides such as Na_2_O and CaO. These modifiers reduce network connectivity and lower the energy barrier for bond cleavage, rendering WGP more susceptible to alkaline attack than crystalline silica phases. Under alkaline activation, hydroxyl ions penetrate the glass surface and progressively cleave siloxane bonds, releasing soluble silicate species into the pore solution [3,6,9,31].

Unlike conventional geopolymer precursors such as fly ash or metakaolin, WGP contains minimal intrinsic aluminium and therefore functions primarily as a silica donor rather than as a complete geopolymer precursor. Its dissolution enriches the pore solution with silicate species that subsequently participate in condensation reactions with aluminium supplied by co-precursors. Consequently, WGP dissolution does not directly generate aluminosilicate oligomers, and stable gel formation remains dependent on the availability of reactive aluminium from complementary materials [33,45].

The rate and extent of WGP dissolution are strongly influenced by particle fineness, glass composition, and activator chemistry. Finer WGP particles exhibit accelerated dissolution due to increased specific surface area and reduced diffusion path lengths, promoting early-stage reaction kinetics and more rapid gel development [3,17]. Glass composition further modulates dissolution behaviour, with soda–lime silicate glasses, which dominate global waste glass streams, dissolving more readily than borosilicate or aluminosilicate glasses due to higher alkali contents, lower network polymerization, and the concurrent release of Ca^2+^ into the pore solution [6,16]. While the calcium contribution from soda–lime glass is typically insufficient to drive hydration-dominated reactions on its own, released Ca^2+^ can influence early reaction kinetics and gel evolution, particularly in blended systems, and should be distinguished from calcium supplied by lime or calcium-rich precursors such as slag [14,18].

Activator chemistry plays a decisive role in controlling WGP dissolution and subsequent geopolymerization. Sodium hydroxide solutions promote rapid depolymerisation of the glass surface by providing high hydroxyl ion concentrations, whereas sodium silicate solutions modify the SiO_2_/Na_2_O ratio of the activating medium and influence the balance between dissolution and condensation processes. Combined NaOH–Na_2_SiO_3_ activator systems have been shown to enhance reaction efficiency in WGP-containing systems by coupling accelerated glass dissolution with improved gel polymerisation [33,54].

While increased activator alkalinity generally accelerates WGP dissolution and early strength development, excessive alkali concentrations can adversely affect pore solution chemistry and long-term stability, contributing to alkali mobility, efflorescence, or durability-related instability. Optimisation of activator concentration and silicate modulus is therefore essential to balance rapid silica release with controlled gel development in WGP-based geopolymer systems [4,33,53].

### 4.6. Gel Formation and Phase Evolution

Silicate species released from waste glass powder (WGP) during alkaline dissolution subsequently participate in geopolymerization reactions through condensation with aluminate and aluminosilicate species supplied by co-precursors such as fly ash, metakaolin, or ground granulated blast furnace slag (GGBS) [3,33,45]. Because WGP contains minimal intrinsic aluminium compared with conventional geopolymer precursors [3,13,45], it generally cannot sustain extensive geopolymer network formation on its own and instead primarily acts as a reactive silica contributor. In blended systems, the dissolved silicate species from WGP modify gel chemistry by enriching the reacting system with additional silica and adjusting the Si/Al ratio of the developing reaction products [33,45].

In low-calcium systems, where reactive aluminium is primarily derived from fly ash or metakaolin, the dominant reaction product is sodium aluminosilicate hydrate (N-A-S-H) gel. This gel consists of a three-dimensional, cross-linked aluminosilicate framework charge-balanced by alkali cations, typically Na^+^ [33,45,55]. In such systems, WGP functions as a supplementary silica source that increases the Si/Al ratio of the developing gel, promoting higher degrees of polymerisation and matrix densification when sufficient aluminium is available. However, excessive replacement of alumina-bearing precursors with WGP can lead to aluminium-deficient systems, limiting network cross-linking and resulting in poorly connected or mechanically weaker gel structures [3,5].

An important characteristic of WGP-containing systems is the delayed contribution of silica arising from the gradual dissolution of residual glass particles [3,9]. This sustained silica release can influence gel evolution beyond early reaction stages, contributing to progressive network reorganisation and densification, provided that aluminium supply remains adequate. Where aluminium becomes limiting, continued silica availability does not translate into further geopolymerization and may instead accumulate in the pore solution or form less integrated reaction products.

In blended systems incorporating calcium-rich precursors such as GGBS or high-calcium fly ash, reaction pathways shift towards the formation of hybrid gel assemblages comprising N-A-S-H and calcium–aluminosilicate hydrate (C-(A)-S-H) phases. These assemblages arise from parallel geopolymerization and slag hydration reactions under alkaline activation [18,20,34,45]. Calcium released primarily from slag, and to a lesser extent from soda–lime glass, accelerates reaction kinetics and contributes to the development of denser but chemically heterogeneous microstructures. Atomistic modelling studies have further shown that Ca^2+^ incorporation within N-A-S-H gel structures can modify local charge-balancing mechanisms and reduce network polymerization, promoting the formation of hybrid gel environments and altering the structural stability of the aluminosilicate framework [56].

Hybrid gel systems in WGP–GGBS blends are frequently associated with enhanced early-age strength and refined pore structures compared with purely N-A-S-H-based matrices. However, these benefits are strongly dependent on calcium availability, activator chemistry, and curing conditions. Excessive calcium contents may suppress geopolymer gel formation and favour hydration-dominated pathways, potentially compromising long-term stability despite initial densification [3,22,45].

Overall, gel formation and phase evolution in WGP-based geopolymer systems are governed by precursor chemistry, aluminium availability, and calcium content rather than by WGP content alone. Moderate WGP incorporation can enhance gel polymerisation and microstructural refinement, whereas high replacement levels without compensating aluminium sources lead to incomplete geopolymerization and suboptimal phase assemblages. These observations reinforce the necessity of balanced precursor design to achieve stable gel chemistry and reliable performance in WGP-containing geopolymer binders [5,20].

### 4.7. Controlling Parameters and Comparative Reactivity of WGP

The reactivity of waste glass powder (WGP) in geopolymer systems is governed by a combination of physical, chemical, and processing-related parameters that collectively distinguish its behaviour from that of conventional geopolymer precursors. Among these, particle fineness represents the most influential controlling factor. Finely ground WGP exhibits enhanced alkaline dissolution and contributes more effectively to reaction kinetics, whereas coarser particles dissolve slowly and may function primarily as micro fillers with limited chemical participation [3,9]. The amorphous nature of glass is a prerequisite for reactivity [31], as crystalline silica phases display markedly lower dissolution rates under alkaline activation.

Glass chemistry further modulates WGP reactivity. Soda–lime silicate glasses, which dominate global waste glass streams, generally dissolve more readily than borosilicate or aluminosilicate glasses due to higher alkali contents and lower network polymerisation [6,16]. While calcium present in soda–lime glass can contribute to early reaction kinetics and hybrid gel formation in blended systems, it may also influence long-term phase stability and durability depending on precursor balance and exposure conditions [45,53].

Processing and curing conditions exert additional control over WGP reactivity. Elevated curing temperatures accelerate glass dissolution and gel formation, particularly in low-calcium systems where ambient curing may be insufficient to activate WGP effectively [54]. Activator composition, including alkali concentration and silicate modulus, governs pore solution chemistry and determines the balance between silica dissolution and condensation reactions, thereby influencing the efficiency and stability of gel formation [33].

In comparison with conventional geopolymer precursors such as fly ash and metakaolin, WGP exhibits fundamentally different reactivity characteristics. Unlike aluminosilicate-rich precursors, WGP contains minimal reactive aluminium and therefore cannot function as a sole geopolymer precursor in most systems. Its primary contribution lies in supplying additional reactive silica, which can enhance reaction kinetics, increase gel polymerisation, and refine microstructure when combined with aluminium-bearing materials [3,5,45].

In calcium-rich systems, particularly those incorporating ground granulated blast furnace slag, WGP contributes to pore refinement and participates in the formation of hybrid N-A-S-H/C-(A)-S-H gel assemblages, often improving mechanical and durability performance at moderate replacement levels [14,18]. However, excessive WGP incorporation may dilute aluminium and calcium availability, leading to incomplete geopolymerization and suboptimal phase development. These observations underscore that the reactivity benefits of WGP are conditional and depend on balanced precursor design, activator chemistry, and curing regime rather than on WGP content alone.

The behaviour of waste glass powder in alkali-activated binders is governed by three interrelated compositional constraints: (i) its silica-rich but Aluminium-deficient composition [3,45], (ii) the formation of hybrid N-A-S-H/C-(A)-S-H gels in Ca-bearing systems [18,20,34], and (iii) the strong dependence of performance on precursor balance and activator chemistry [3,5,33]. These factors control reaction completeness, gel assemblage, and pore structure development. Consequently, the performance of WGP cannot be interpreted solely as a function of replacement level but must be considered in relation to Aluminium availability, calcium content, and overall system design.

**Figure 3:**
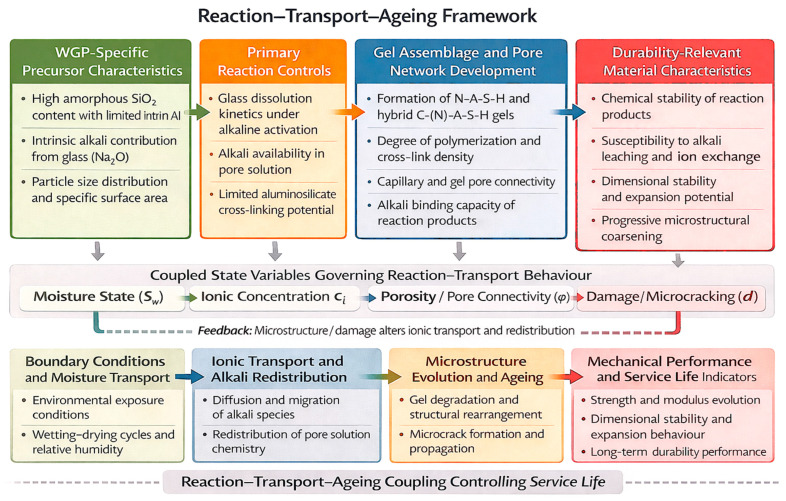
Reaction–transport–ageing framework describing durability evolution in waste glass powder–based geopolymer binders. The framework links precursor dissolution and gel formation (reaction domain) to pore connectivity and ionic migration (transport domain), which interact with microstructural ageing processes to determine long-term mechanical performance and service life.

While Section 4.1, Section 4.2, Section 4.3, Section 4.4, Section 4.5, Section 4.6 and Section 4.7 discuss the individual factors governing WGP reactivity and gel development, durability performance emerges from the interaction of these processes with transport and ageing phenomena rather than from isolated mechanisms. To synthesise the relationships between WGP precursor characteristics, reaction controls, gel assemblage, and long-term performance, a reaction–transport–ageing framework is proposed (Figure 3). The reaction mechanisms discussed above determine the phase assemblage, gel chemistry, and pore network architecture that ultimately control fluid ingress, ion mobility, and long-term microstructural stability. Durability performance in WGP-based geopolymer systems, therefore, cannot be interpreted independently of reaction chemistry but must be understood as the coupled evolution of reaction, transport, and ageing processes. This mechanistic interdependence forms the basis of the reaction–transport–ageing (R–T–A) framework applied in Section 5.

## 5. Durability Evolution Through Reaction–Transport–Ageing Coupling

Durability in WGP-based geopolymer systems does not arise from isolated performance indicators but from the coupled evolution of reaction chemistry, transport processes, and ageing phenomena. Within the proposed reaction–transport–ageing (R–T–A) framework (Figure 3), durability outcomes emerge through three interdependent stages: (i) reaction-controlled gel formation and phase assemblage development, (ii) transport-controlled ingress, leaching, and moisture migration governed by pore connectivity, and (iii) time-dependent gel reorganisation and alkali redistribution that define long-term stability.

In this context, WGP influences durability first through its dissolution behaviour and contribution to gel chemistry, which determine the initial pore structure and phase stability. These reaction-controlled characteristics subsequently regulate transport properties, including ion diffusion and moisture movement, which in turn interact with ageing mechanisms such as decalcification, gel restructuring, and alkali mobility [33,53]. Durability performance is therefore an emergent property of this coupled evolution rather than a direct consequence of WGP content alone.

Unlike OPC systems, where degradation is frequently governed by portlandite dissolution and expansive secondary phase formation, alkali-activated binders rely on the chemical and structural stability of aluminosilicate and hybrid calcium–aluminosilicate gels [32,33]. In WGP-containing systems, gel composition, degree of polymerisation, pore solution chemistry, and pore network connectivity collectively define how reaction, transport, and ageing interact under environmental exposure.

The following subsections interpret specific durability domains explicitly through this R–T–A coupling framework rather than as isolated performance metrics.

A structured synthesis of reported durability outcomes associated with WGP inclusion in geopolymer and alkali-activated binder systems is presented in Table 5, organised across key durability domains and differentiated between low-calcium (e.g., FA/MK-dominated) and calcium-rich (e.g., WGP–GGBS, FA–GGBS) systems. This framework highlights that WGP does not exert a uniform durability effect; instead, its influence is system-specific and emerges from coupled chemical and microstructural mechanisms rather than from the presence of glass alone.

Across chemical resistance domains, including sulphate and acid exposure, WGP incorporation has been reported to be generally stable or moderately beneficial when the reaction proceeds sufficiently and pore connectivity is reduced [5,27,57]. In low-calcium systems, the stability of N-A-S-H-type gels and reduced availability of calcium phases underpin favourable resistance trends, whereas in Ca-rich blends, durability becomes more sensitive to slag content and exposure severity due to potential decalcification or alteration of hybrid C(N)-A-S-H gels [14,32,33]. These trends, summarised in Table 5, underline that WGP-related improvements in chemical resistance are contingent on balanced Si/Al ratios and controlled calcium availability rather than on glass content alone.

Transport-related durability indicators, including water absorption, sorptivity, and chloride migration resistance, show more consistent benefits at moderate WGP replacement levels, particularly when fine WGP contributes to pore refinement through combined filler effects and enhanced gel formation [3,5]. As synthesised in Table 5, both low-Ca and Ca-rich systems can exhibit reduced permeability when WGP fineness and activator chemistry are optimised; however, excessive WGP replacement or aluminium limitation can lead to incomplete geopolymerization and increased connected porosity [22,58]. This explains the variability reported in chloride transport performance, especially in systems cured under ambient conditions [14,20].

Serviceability-related durability concerns, such as efflorescence and alkali leaching, remain relevant for WGP-containing systems, particularly under high-alkali activation and incomplete reaction conditions. As indicated in Table 5, the immobilisation of alkalis within N-A-S-H or hybrid gels is critical; where aluminium availability or curing is insufficient, WGP-derived silica alone does not prevent alkali mobility, leading to surface salt formation and leaching [5,33,53,59].

The role of WGP in alkali–silica reaction (ASR)-type expansion in alkali-activated materials differs fundamentally from its behaviour in OPC systems. While fine WGP is widely reported as ASR-mitigating in cementitious binders, evidence in geopolymer systems remains mixed and highly dependent on activator chemistry, aluminium availability, and pore solution composition [4,60,61]. As summarised in Table 5, fine WGP typically dissolves and participates in binder formation rather than acting as a reactive aggregate; however, high alkali concentrations combined with insufficient aluminium can promote silica-rich reaction products and potential volumetric instability [45,62].

Shrinkage behaviour and long-term ageing further illustrate the trade-offs associated with WGP-induced densification. While pore refinement can reduce permeability, it may simultaneously increase drying or autogenous shrinkage through enhanced capillary stresses, particularly in silica-rich and Ca-containing systems [5,58]. Long-term durability data remain limited, and as highlighted in Table 5, the coupled effects of leaching, gel reorganisation, and cyclic environmental exposure represent a key research gap for WGP-based geopolymer binders [29,32,51].

### 5.1. Chemical Durability: Sulphate and Acid Resistance

As illustrated in Figure 3, sulphate and acid resistance emerge from the interaction between reaction-controlled gel chemistry and transport-controlled ion ingress. Chemical resistance to sulphate and acid-rich environments in WGP-based geopolymer systems is best interpreted through the reaction–transport–ageing (R–T–A) framework rather than as an isolated material property.

#### 5.1.1. Reaction-Controlled Stage

At the reaction stage, intrinsic chemical stability is governed primarily by gel chemistry and calcium availability. In low-calcium systems dominated by N-A-S-H gel, the absence of portlandite and the limited formation of expansive calcium aluminate or calcium sulphate phases reduce susceptibility to sulphate-induced expansion and acid-driven decalcification [31,32,33]. The highly cross-linked aluminosilicate framework characteristic of N-A-S-H gels provides improved structural persistence under moderate sulphate and acidic environments compared to calcium-rich hydrates [45,58].

In such systems, adequately dissolved WGP functions as a reactive silica source, increasing the Si/Al ratio and promoting higher polymerisation of the aluminosilicate network [52]. When aluminium supply remains sufficient to maintain charge balance, this reaction-controlled densification enhances intrinsic resistance to chemical attack. Experimental studies on WGP-modified fly ash and metakaolin systems report stable to moderately improved sulphate resistance under sodium sulphate exposure conditions, particularly when replacement levels do not induce aluminium dilution [27,57].

In contrast, Ca-rich WGP–GGBS systems generate hybrid N-A-S-H/C-(A)-S-H assemblages [18]. Moderate calcium contents can accelerate reaction kinetics and refine early microstructure. However, excessive calcium shifts the reaction pathway toward C-(A)-S-H-rich assemblages that are more vulnerable to decalcification under acidic environments and to destabilisation under magnesium sulphate attack [14,58]. Thus, chemical durability at the reaction stage is controlled by phase assemblage stability and Ca/Si balance rather than by WGP incorporation alone.

Importantly, soda-lime WGP introduces not only silica but also alkali species, which may increase pore solution alkalinity. While this can enhance early dissolution and polymerisation, it may also contribute to alkali mobility and efflorescence risk if not structurally immobilised within the gel network [53,59]. Therefore, reaction-controlled durability depends on balanced dissolution, adequate aluminium availability, and controlled calcium content.

#### 5.1.2. Transport-Controlled Stage

As exposure progresses, sulphate and acid resistance become increasingly transport-controlled. Ion ingress, leaching, and reaction front advancement are governed by pore connectivity and tortuosity established during the reaction stage [32]. Finely ground WGP can contribute to pore refinement through both silica enrichment and micro-filler effects, reducing connected porosity and limiting sulphate or proton penetration when geopolymerization is sufficiently complete [18,20].

Studies on WGP-containing alkali-activated slag and fly ash systems demonstrate reduced chloride and ion diffusion coefficients when glass is optimally incorporated, indicating transport limitation through densified matrices [20,52]. However, excessive WGP replacement or insufficient aluminium supply may result in incomplete reaction and increased connected porosity, offsetting intrinsic gel stability advantages. Under such conditions, sulphate ingress and acid attack become governed by permeability rather than chemistry alone.

Thus, chemical resistance at this stage reflects the interaction between reaction completeness and transport limitation, not simply the presence of glass powder.

#### 5.1.3. Ageing-Controlled Evolution

Under prolonged exposure, ageing mechanisms such as decalcification, gel alteration, secondary phase formation, and alkali migration determine long-term performance. In low-calcium N-A-S-H-dominated systems, the three-dimensional aluminosilicate network generally exhibits greater structural persistence under sodium sulphate and moderate acid exposure, provided that transport pathways remain limited [27,57].

In Ca-rich hybrid systems, prolonged exposure to strong acids can induce progressive decalcification of C-(A)-S-H phases, resulting in microstructural weakening despite initially dense matrices [33,58]. Similarly, magnesium sulphate environments may destabilise calcium-rich hydrates, leading to phase transformation and mechanical degradation [14]. Ageing performance, therefore, depends on the long-term thermodynamic stability of the formed phases rather than short-term strength retention.

Additionally, alkali mobility associated with WGP dissolution may contribute to efflorescence and surface precipitation phenomena during ageing if excess sodium remains unbound [53,59]. These processes do not necessarily indicate immediate structural failure but reflect evolving pore solution chemistry and ion transport dynamics.

Overall, sulphate and acid resistances in WGP-containing geopolymer binders emerge from the coupled interaction of reaction-controlled gel chemistry, transport-controlled ion ingress, and ageing-driven phase evolution. WGP enhances chemical durability only when its dissolution contributes to stable gel formation, maintains aluminium balance, and supports pore refinement without generating alkali mobility or transport vulnerabilities. The system-dependent trends summarised in Table 5 therefore reflect differences in R–T–A balance rather than intrinsic inconsistency in WGP behaviour.

### 5.2. Transport-Related Durability: Water Absorption, Sorptivity, and Chloride Resistance

Within the reaction–transport–ageing (R–T–A) framework, transport represents the governing pathway through which environmental exposure interacts with the reaction products formed during activation. While reaction controls gel chemistry and initial pore structure, long-term durability under chloride, moisture, or multi-ion exposure is fundamentally transport-controlled [32,33]. In alkali-activated systems, permeability and ion diffusivity are dictated primarily by pore size distribution, connectivity, and gel continuity rather than by bulk compressive strength alone [45,58].

#### 5.2.1. Reaction–Transport Linkage

Transport properties in WGP-based geopolymer systems are determined by the pore structure established during the reaction stage. Finely ground WGP supplies reactive silica that, when balanced by adequate aluminium availability, increases gel polymerisation and promotes pore refinement [5,6]. Microstructural investigations of WGP-modified binders report reduced capillary porosity and improved matrix densification when glass is sufficiently reactive and well dispersed [18,19,52].

This reaction-driven densification reduces connected capillary pores and lowers water absorption and sorptivity. Experimental studies on WGP-containing cementitious and alkali-activated systems consistently report reductions in water absorption and permeability at moderate replacement levels, particularly when glass fineness enhances dissolution kinetics [5,18].

However, transport performance cannot be interpreted independently of reaction completeness. Excessive WGP replacement at the expense of alumina-bearing precursors may dilute reactive aluminium, limiting network formation and increasing connected porosity despite local filler-induced densification [5,22]. Under such conditions, apparent short-term densification does not translate into durable transport resistance. Thus, transport behaviour reflects the balance between silica enrichment and structural network integrity established during activation.

#### 5.2.2. Chloride Ingress as a Transport-Dominated Mechanism

Chloride resistance in WGP-containing geopolymer systems is primarily governed by pore connectivity and tortuosity rather than by extensive chemical chloride binding, particularly in low-calcium systems where AFm-type binding phases are limited [45]. Reduced chloride migration and diffusion coefficients have been reported in WGP-modified alkali-activated slag/fly ash systems under controlled curing, attributable to refined microstructure and reduced connected porosity [14,20]. It should be noted that much of the available chloride transport evidence derives from paste or mortar specimens tested under controlled laboratory curing conditions. The extent to which these trends translate to structural geopolymer concretes, where aggregate interaction and field curing influence pore connectivity, remains an active research question [5,33,45].

In Ca-rich blends, hybrid N-A-S-H/C-(A)-S-H gels may further reduce permeability when calcium content contributes to matrix densification without generating microcracking or phase instability [58]. However, chloride durability remains transport-limited once initial gel formation is complete. Within the R–T–A perspective, WGP influences chloride resistance indirectly: it modifies reaction chemistry, which shapes pore structure, which governs ionic transport.

This distinction is critical. Chloride resistance improvement, therefore, reflects reduced diffusivity associated with pore refinement and increased tortuosity rather than strong chemical chloride binding. Unlike OPC systems, where AFm phases can chemically immobilise chlorides, low-calcium alkali-activated binders typically exhibit limited binding capacity, and improved durability is primarily a transport-controlled effect [20,45].

#### 5.2.3. Transition Toward Ageing Control

Over extended exposure periods, transport processes interact with ageing mechanisms. Sustained moisture and ion ingress may promote alkali redistribution, efflorescence, gel alteration, or gradual decalcification in Ca-rich systems, particularly when alkali mobility remains high [53,59]. Consequently, transport-related durability cannot be separated from ageing evolution, as the ingress pathways established during reaction define the long-term stability trajectory.

In the context of ASR-type behaviour, the role of WGP must be interpreted cautiously. Finely ground glass incorporated as a reactive precursor within a stable geopolymer network generally dissolves and participates in gel formation rather than behaving as a reactive aggregate [4,5,45]. Consequently, expansive behaviour observed in some alkali-activated systems may reflect silica dissolution, alkali mobility, or gel reorganisation rather than true alkali–silica reaction [60,61,62].

In alkali-activated materials, classification of ASR should therefore be supported by combined evidence including measurable expansion, petrographic identification of silica-reaction products at aggregate interfaces, and chemical characterisation of silica-rich gels. Many reported “ASR- like” phenomena in WGP systems lack this diagnostic combination and may instead reflect broader reaction–transport instabilities associated with residual reactive silica and high pore solution alkalinity [60,61,62]. Thus, ASR-type expansion in WGP systems reflects incomplete reaction or transport-facilitated alkali mobility rather than intrinsic glass reactivity alone.

Overall, improvements in water absorption, sorptivity, and chloride resistance reported for WGP-containing systems emerge only when reaction-controlled gel development produces a stable, continuous, and well-connected matrix. Transport resistance is therefore conditional on precursor balance [63], WGP fineness, activator chemistry, and curing regime [5,20,22]. The variability summarised in Table 5 reflects differences in R–T–A balance rather than inconsistency in WGP performance. As illustrated in Figure 3, transport durability is the functional bridge between early reaction chemistry and long-term ageing stability.

### 5.3. Alkali–Silica Reaction (ASR): Mitigation Versus Risk in WGP-Based Geopolymer Systems

Within the reaction–transport–ageing (R–T–A) framework, expansion phenomena associated with silica dissolution in WGP-based geopolymer systems should be interpreted as coupled instabilities rather than as a single reaction mechanism. Expansion may occur when silica dissolution during activation (reaction), sustained alkali and moisture mobility (transport), and time-dependent gel swelling or phase transformation (ageing) interact under unfavourable chemical and microstructural conditions [32,45]. These interacting processes control whether dissolved silica remains chemically incorporated within the geopolymer network or contributes to volumetric instability over time. Consequently, expansion behaviour in alkali-activated systems cannot be inferred directly from classical OPC-based interpretations of alkali–silica reaction.

In alkali-activated systems, the term “ASR-type behaviour” is often used broadly to describe expansion phenomena associated with silica dissolution or alkali–moisture interactions, even when classical aggregate–gel reactions are not observed. True alkali–silica reaction involves the formation of expansive silica gel at reactive aggregate–binder interfaces, accompanied by measurable expansion. In contrast, many expansion phenomena reported in alkali-activated binders appear to arise from silica dissolution, gel reorganisation, or osmotic swelling within the binder matrix rather than from aggregate-driven ASR. Diagnostic confirmation of true ASR therefore requires combined evidence, including measurable expansion, petrographic identification of reaction products at aggregate interfaces, and chemical characterisation of silica-rich gels using techniques such as SEM–EDS [45,60,61,62]. Distinguishing between these mechanisms is essential when interpreting durability behaviour in WGP-containing geopolymer systems.

#### 5.3.1. Reaction-Controlled Stage

At the reaction stage, finely ground WGP behaves fundamentally differently from reactive glass aggregate in OPC systems. The high surface area of fine WGP promotes rapid dissolution and incorporation of silicate species into N-A-S-H or hybrid C(N)-A-S-H gels rather than the formation of discrete expansive alkali–silica gels [5,9]. Particle size plays a decisive role: finely ground glass tends to react pozzolanically or geopolymerically, whereas coarse glass particles behave as reactive aggregates in OPC environments [9].

In alkali-activated matrices, the absence of portlandite and the dominance of aluminosilicate networks modify silica–alkali equilibria relative to OPC systems [32,45]. Aluminium incorporation into the gel structure provides charge-balancing sites that chemically immobilise dissolved silica within a cross-linked framework, thereby limiting the accumulation of free silica species that could otherwise participate in expansive reactions [4].

When aluminium availability is sufficient, and activator dosage is controlled, WGP-derived silica becomes structurally integrated within the geopolymer network. Under these conditions, the reaction stage favours network formation and chemical incorporation of silica rather than the accumulation of free alkali–silica gel. Experimental studies, therefore, often report negligible or reduced expansion in fine WGP-modified systems when incorporated within chemically balanced alkali-activated matrices [27,60]. However, whether such stability is maintained depends on subsequent transport and ageing processes, which control alkali mobility, moisture availability, and long-term gel evolution.

#### 5.3.2. Transport-Controlled Stage

ASR-type expansion risk increases when transport processes permit sustained alkali and moisture availability. Even if initial gel formation is chemically stable, elevated alkali concentrations combined with continuous moisture ingress can increase dissolved silicate levels in the pore solution. Where silica release exceeds the incorporation capacity of the gel network, silica-rich products may accumulate locally within the pore structure [18,20].

Recent investigations demonstrate that alkali concentration, pore connectivity, and moisture exposure strongly influence expansion behaviour in alkali-activated glass systems [60,61,62]. These findings indicate that expansion phenomena associated with WGP-containing geopolymers are transport-enabled rather than purely reaction-driven. Pore connectivity, alkali mobility, and moisture gradients determine whether dissolved silica remains chemically immobilised within the geopolymer framework or contributes to localised swelling and volumetric instability.

Within the R–T–A perspective, transport processes therefore regulate the conditions under which silica dissolution products interact with the evolving gel network. Limiting connected porosity and alkali mobility through microstructural densification can reduce the likelihood of such instabilities, although long-term behaviour remains dependent on the coupled effects of transport and ageing.

#### 5.3.3. Ageing-Controlled Evolution

Over extended exposure periods, ageing processes determine whether early-stage microstructural stability is maintained. Alkali redistribution, gel reorganisation, and phase transformation may progressively modify pore solution chemistry and internal restraint conditions. In Ca-rich WGP–slag systems, hybrid C-(A)-S-H/N-A-S-H gels can undergo gradual chemical modification, including partial decalcification or structural rearrangement, which may influence volumetric stability under sustained moisture and alkali exposure [18,61].

Moderate calcium contents may contribute to matrix densification and reduced pore connectivity, thereby limiting transport pathways for alkali and moisture. However, excessive calcium may produce chemically less stable phases that become vulnerable to alteration or decalcification under aggressive exposure conditions, increasing susceptibility to microcracking and volumetric instability [14,61]. Consequently, long-term expansion behaviour is governed primarily by the evolving stability of the gel assemblage rather than by initial silica reactivity alone.

Within the reaction–transport–ageing framework, expansion phenomena associated with WGP-containing alkali-activated binders therefore reflect the dynamic balance between reaction-controlled silica incorporation, transport-controlled alkali and moisture availability, and ageing-driven gel evolution. Fine WGP typically contributes to stable network formation when incorporated within chemically balanced systems with controlled alkali dosage and adequate aluminium availability [60,62]. Conversely, excessive WGP replacement, high activator concentrations, or aluminium limitation may disrupt this balance and increase the likelihood of volumetric instability.

The variability reported across experimental studies thus reflects differences in reaction–transport–ageing balance rather than intrinsic glass reactivity. Expansion risk in WGP-based geopolymer binders is therefore system-dependent and cannot be inferred solely from silica content or by direct analogy with OPC-based alkali–silica reaction behaviour.

### 5.4. Long-Term Ageing and Coupled Degradation Mechanisms

As illustrated in Figure 3, ageing represents the temporal convergence of reaction and transport processes. Within the reaction–transport–ageing (R–T–A) framework, ageing is not an independent degradation category but the cumulative outcome of reaction-controlled phase assemblage interacting with transport-controlled environmental exposure over time [32,33]. Long-term performance, therefore, depends on both intrinsic gel stability and sustained control of ingress pathways.

#### 5.4.1. Reaction–Ageing Linkage

Ageing in WGP-based geopolymer systems begins with the stability and adaptability of the reaction products formed during activation. In silica-rich systems, residual WGP particles may continue to dissolve beyond early curing stages, particularly when particle size distribution includes partially reacted glass cores [5,18]. This delayed dissolution can supply additional silicate species to the pore solution.

When aluminium availability remains sufficient, and charge balance is maintained, such continued dissolution may promote progressive gel reorganisation, increased polymerisation, and gradual pore refinement [52]. This reaction–ageing coupling can enhance long-term transport resistance through continued matrix densification, particularly in well-proportioned low-calcium systems.

However, if aluminium supply is insufficient or activator dosage is excessive, delayed silica release may exceed incorporation capacity. Under such conditions, ageing does not lead to densification but may instead increase pore solution alkalinity and destabilise local equilibrium. Therefore, ageing behaviour is governed primarily by the structural resilience of the initial gel assemblage rather than by WGP presence alone.

#### 5.4.2. Transport–Ageing Interaction

Transport processes define the environmental drivers of ageing. Sustained moisture ingress, alkali mobility, and external ion penetration modify pore solution chemistry and may trigger gradual gel alteration. In Ca-rich systems, hybrid C-(A)-S-H/N-A-S-H assemblages may undergo progressive decalcification under acidic or sulphate-rich exposure, altering mechanical integrity over time [32,33,58]. Decalcification reduces cross-link density and may increase susceptibility to microcracking under sustained transport-driven attack.

Even in low-calcium systems, prolonged alkali redistribution can affect dimensional stability and promote surface precipitation phenomena such as efflorescence when excess sodium remains mobile [53,59]. Efflorescence does not necessarily indicate immediate structural failure, but it reflects ongoing alkali migration and pore solution evolution that may influence long-term durability.

Thus, ageing is transport-enabled: the pore connectivity and tortuosity established during reaction determine the degree to which environmental exposure influences gel stability and volumetric behaviour.

#### 5.4.3. Microstructural Evolution and Analytical Interrogation

Ageing-related gel reorganisation must be assessed using techniques sensitive to aluminosilicate network evolution. Fourier-transform infrared spectroscopy (FTIR) provides insight into Si–O–T (T = Si or Al) band shifts associated with polymerisation changes and structural rearrangement in alkali-activated materials [31,33]. Shifts toward lower wavenumbers are often interpreted as changes in polymerization or altered Si/Al environments.

Thermogravimetric analysis (TGA), while useful for assessing dehydration, carbonation, and bound-water changes, does not directly resolve network restructuring. It captures mass-loss phenomena associated with phase transformations or water release [33]. Therefore, differentiating reaction-driven densification from exposure-driven degradation requires mechanistically aligned characterisation, ideally integrating FTIR with complementary techniques such as SEM-EDS or NMR.

Without such alignment, long-term behaviour may be misinterpreted as degradation when it reflects ongoing structural maturation.

#### 5.4.4. Integrated Ageing Outcome

Ultimately, the long-term durability of WGP-containing geopolymer binders emerges from the stability of reaction products under transport-driven exposure. Sustained silica release from residual glass may contribute positively to matrix refinement when gel chemistry is balanced and alkali mobility is controlled [18,52]. Conversely, under aggressive, cyclic, or high-alkali environments, ageing may be dominated by leaching, alkali redistribution, decalcification, or gel alteration processes that progressively compromise structural integrity [58].

The variability reported across long-term studies reflects differences in R–T–A balance rather than inherent unpredictability in WGP behaviour [4,57]. However, the limited availability of extended-duration field data for WGP-based alkali-activated systems remains a critical gap. Much of the current evidence derives from paste or mortar specimens exposed to accelerated laboratory conditions, which may not fully capture the coupled ageing–transport phenomena occurring in structural geopolymer concretes over service-life timescales [57].

Robust service-life prediction, therefore, requires exposure regimes that integrate reaction completeness, transport properties, and environmental cycling rather than relying solely on short-term strength or permeability indicators.

Durability evolution in alkali-activated systems also has implications for structural monitoring and maintenance strategies. In-service infrastructure increasingly relies on data-driven warning systems that integrate environmental and structural response indicators, such as temperature, displacement, or vibration monitoring. For example, recent studies on bridge infrastructure have demonstrated warning methodologies based on temperature–displacement monitoring and multi-rate data fusion to detect early structural performance changes under environmental loading [64,65]. Linking durability assessment with such monitoring frameworks may support earlier identification of material degradation and improve service-life management of geopolymer-based structural components. Structural health monitoring systems are widely applied for the early detection of damage and condition assessment in bridge cables and other critical components of long-span bridges [66]. Within the reaction–transport–ageing perspective, monitoring variables such as temperature, displacement, and environmental exposure can provide indirect indicators of transport processes and progressive material degradation.

### 5.5. Contextual Benchmarking Against OPC-Based Systems

Contextual comparison with ordinary Portland cement (OPC) systems is essential for interpreting the durability significance of WGP-based alkali-activated binders. Within the reaction–transport–ageing (R–T–A) framework, the fundamental distinction between OPC and geopolymer systems lies in the dominant reaction products that govern subsequent transport behaviour and ageing evolution [32,33].

#### 5.5.1. Reaction-Stage Contrast

In OPC concretes, durability is strongly influenced by the stability of calcium-rich hydration products, particularly C–S–H and portlandite. Portlandite buffers pore solution chemistry but also introduces phases susceptible to sulphate attack and acid-driven decalcification [32]. The dissolution of Ca (OH)_2_ under aggressive exposure contributes to pore solution changes and matrix destabilisation, often accompanied by progressive decalcification of C–S–H in acidic or sulphate-rich environments.

In contrast, alkali-activated binders incorporating WGP rely primarily on aluminosilicate (N-A-S-H) or hybrid C(N)-A-S-H gels formed during reaction [33,45]. The absence of discrete portlandite phases fundamentally alters chemical stability pathways. Durability control shifts from calcium hydroxide buffering to the structural integrity and cross-link density of the aluminosilicate gel network.

This does not imply inherent superiority. Rather, it reflects different thermodynamic and kinetic controls governing phase stability under exposure.

#### 5.5.2. Transport-Stage Contrast

Transport behaviour in OPC systems is governed by capillary pore connectivity, degree of hydration, and, in blended systems, secondary pozzolanic reactions that refine microstructure. Supplementary cementitious materials in OPC systems can significantly reduce permeability and chloride ingress when properly proportioned.

In WGP-containing geopolymers, transport properties are more directly linked to reaction completeness and gel continuity established during activation. When reaction is well balanced, reduced calcium hydroxide availability and refined pore structure may enhance resistance to sulphate and acid exposure relative to conventional OPC systems lacking supplementary cementitious materials [27]. However, transport-related indicators such as chloride ingress resistance are not uniformly superior.

Chloride diffusion coefficients reported for WGP-modified alkali-activated systems are often comparable to those of well-designed OPC concretes incorporating SCMs rather than consistently lower [14,20]. Performance is highly sensitive to curing regime, activator concentration, and precursor balance. Therefore, transport-stage advantages in WGP systems are conditional rather than intrinsic.

#### 5.5.3. Ageing-Stage Contrast

Ageing mechanisms also diverge. In OPC systems, long-term degradation frequently involves portlandite depletion, decalcification of C–S–H, and formation of expansive secondary products under sulphate or carbonation exposure. These processes are strongly calcium-dependent.

In alkali-activated WGP systems, ageing is governed by the stability and reorganisation of aluminosilicate or hybrid gels under transport-driven exposure [33,58]. Decalcification may occur in Ca-rich blends, but the mechanistic pathway differs from OPC systems due to the absence of a discrete portlandite reservoir and the different structural role of calcium within hybrid C(N)-A-S-H gels. Alkali redistribution and gradual gel modification may influence dimensional stability and serviceability, yet the degradation sequence is chemically distinct from classical OPC deterioration.

Alkali–silica reaction (ASR) behaviour further illustrates these contrasts. In OPC concretes, finely ground waste glass may mitigate expansion through pozzolanic alkali consumption, while coarse glass aggregate may trigger expansive gel formation. In alkali-activated matrices incorporating WGP, ASR-type behaviour reflects the balance between silica incorporation, alkali mobility, and gel evolution within the R–T–A framework [60,61,62]. Expansion may be suppressed in chemically balanced systems or exacerbated under high alkali dosage and sustained moisture exposure. Direct analogy with OPC-based ASR mechanisms is therefore inappropriate.

Overall, benchmarking against OPC systems demonstrates that WGP-based geopolymers do not exhibit unconditional durability superiority but instead operate under fundamentally different reaction–transport–ageing controls. Meaningful comparison requires alignment of curing regimes, exposure conditions, alkali dosage, and specimen scale. Without such alignment, apparent performance differences may reflect differences in reaction completeness or transport limitation rather than intrinsic material behaviour.

### 5.6. Reinforcement Compatibility and Corrosion Considerations

The reliable structural application of WGP-based alkali-activated binders also depends on their compatibility with embedded steel reinforcement. In conventional OPC concretes, corrosion protection is primarily provided by the highly alkaline pore solution generated during hydration, which promotes the formation of a passive oxide film on steel surfaces. Alkali-activated systems generally exhibit comparable or higher pore-solution alkalinity due to dissolved activator species, which can likewise sustain passive film formation under favourable conditions [67,68].

Within the reaction–transport–ageing framework, reinforcement durability is influenced by the same coupled mechanisms governing binder performance. Reaction-controlled gel chemistry determines pore solution composition and the stability of passive films, transport processes regulate chloride ingress and oxygen availability at the steel interface, and ageing processes influence long-term pore structure and alkali redistribution. Experimental studies on reinforced alkali-activated concretes have reported corrosion behaviour comparable to well-designed OPC systems when dense microstructures limit chloride transport [69]. However, differences in chloride binding capacity and pore solution chemistry mean that corrosion resistance in low-calcium alkali-activated binders may depend more strongly on transport limitation than on chemical chloride immobilisation.

Consequently, reinforcement durability in WGP-containing geopolymer systems should be evaluated using integrated durability frameworks that account for reaction chemistry, transport properties, and long-term environmental exposure.

Across the durability domains discussed above, the same compositional constraints repeatedly emerge: aluminium availability controls geopolymer network formation, calcium governs hybrid gel development, and activator chemistry determines reaction completeness. These coupled factors explain why WGP performance varies widely between studies and reinforce the need for durability-informed mix design.

## 6. Research Gaps and Future Directions

The research gaps identified below can be interpreted within the reaction–transport–ageing (R–T–A) framework introduced in Section 5. Current limitations arise from incomplete understanding of reaction-controlled gel development, insufficient quantification of transport processes under realistic exposure, and scarce long-term ageing data linking microstructural evolution to service-life performance.

Despite increasing interest in waste glass powder (WGP) as a sustainable precursor or supplementary component in geopolymer binders, several unresolved research gaps continue to limit its reliable structural application. A central limitation across the literature is the predominance of short- to medium-term mechanical and durability assessments, with most data summarised in Table 5 derived from curing ages of approximately 28–90 days and relatively short exposure periods. Such timeframes are insufficient to capture ageing-stage evolution within the reaction–transport–ageing framework governing long-term performance, particularly under sustained chemical exposure, moisture transport, and alkali redistribution. Extended ageing studies addressing the interaction between chemical exposure, transport processes, alkali mobility, and microstructural evolution remain scarce, particularly for WGP-based systems subjected to realistic service conditions and durations extending beyond conventional laboratory timescales.

A further critical gap arises from the variability of waste glass composition. Most studies rely on locally sourced soda–lime glass with limited reporting of compositional variability, impurity content, or amorphous fraction, despite the inherent heterogeneity of post-consumer glass streams. While comprehensive control of glass chemistry is often impractical, insufficient characterisation hinders cross-study comparability and obstructs the development of transferable mix design strategies. Future research should therefore focus on systematic characterisation and classification of waste glass powders, explicitly linking broad compositional descriptors (e.g., CaO–Na_2_O–SiO_2_ balance, amorphous content, and fineness) and dissolution behaviour to reaction kinetics, gel structure, and durability outcomes, rather than treating WGP as a chemically uniform material. Insufficient compositional characterisation limits predictive control of reaction kinetics and gel assemblage formation.

Alkali–silica reaction (ASR)-type behaviour in WGP-containing geopolymer systems remains particularly poorly resolved. While fine WGP is frequently reported to mitigate expansion, the mechanistic boundaries separating expansive ASR from non-expansive silica-driven degradation processes are not well defined. In alkali-activated matrices, silica released from WGP may either participate beneficially in binder gel formation or contribute to instability through altered pore solution chemistry, alkali redistribution, or gel reorganisation without the formation of a discrete expansive ASR gel. Key thresholds related to alkali dosage, aluminium availability, calcium content, and particle size remain system-specific and inconsistently reported. There is a clear need for controlled, mechanism-oriented studies combining expansion measurements with pore solution analysis and microstructural characterisation to distinguish swelling-driven ASR from transport- or chemistry-driven silica-related degradation phenomena in alkali-activated systems. This behaviour likely reflects a coupled reaction–transport instability rather than a clearly defined expansive ASR mechanism.

The dominance of laboratory-scale curing and exposure regimes represents a major limitation across the existing literature on WGP-based alkali-activated binders. Many reported durability improvements are derived from paste or mortar specimens cured under elevated temperatures or tightly controlled environments, which do not reflect ambient curing, humidity fluctuations, temperature cycling, or combined mechanical and environmental loading encountered in practice [32,45]. Translation of laboratory-scale trends to concrete-scale applications, therefore, remains uncertain, particularly with respect to long-term transport behaviour, dimensional stability, and coupled degradation mechanisms under realistic service conditions. Long-term field exposure studies and performance-based durability testing are essential to validate laboratory findings and establish confidence in the robustness of WGP-based geopolymer binders for structural use.

Practical implementation challenges include variability in waste glass composition, sensitivity of reaction kinetics to curing conditions, reinforcement compatibility in highly alkaline pore solutions, and the absence of standardised durability testing frameworks specific to alkali-activated materials [33,58]. Addressing these constraints is essential before WGP-based geopolymer binders can be reliably benchmarked against OPC concretes in structural applications. Future research should therefore prioritise long-term, concrete-scale durability assessments under realistic exposure conditions, alongside mechanism-based design approaches that explicitly link reaction chemistry, gel evolution, and transport behaviour.

Finally, durability optimisation is rarely integrated with sustainability assessment, despite durability being a prerequisite for meaningful sustainability gains. Although WGP offers clear environmental benefits through waste valorisation and reduced reliance on virgin raw materials, these benefits are contingent on achieving sufficient long-term durability and service life. Uncertainties in degradation behaviour, ageing, and serviceability directly undermine life-cycle performance by shortening functional lifespan and increasing repair or replacement demands. Comprehensive assessments linking durability-driven service life predictions with life-cycle environmental impacts are therefore required to ensure that sustainability claims for WGP-based geopolymer binders are both technically robust and environmentally defensible.

Integrating service-life modelling within a reaction–transport–ageing framework is therefore essential to ensure that durability performance and environmental sustainability are evaluated on a mechanistically consistent basis. Addressing these research gaps will enable more reliable interpretation of WGP performance across the reaction, transport, and ageing stages that collectively govern long-term durability.

## 7. Conclusions

This review examined the role of waste glass powder (WGP) in alkali-activated and geopolymer binder systems from a mechanistic perspective by integrating reaction chemistry, gel evolution, transport behaviour, and long-term ageing processes. Using a reaction–transport–ageing (R–T–A) framework, the analysis clarifies how precursor balance, calcium availability, and mix design collectively govern the performance variability reported across WGP-based systems. In most alkali-activated binders, WGP functions primarily as a reactive silica contributor because its relatively low intrinsic aluminium content requires combination with alumina-bearing precursors such as fly ash, slag, or metakaolin to sustain stable aluminosilicate network formation. When appropriately proportioned, these blended systems enhance reaction kinetics, gel polymerisation, and microstructural development, whereas poorly balanced mixtures may result in incomplete geopolymerization and reduced performance.

The nature and stability of the resulting gel assemblage are strongly influenced by calcium availability. Low-calcium systems are typically dominated by N-A-S-H gel networks, while calcium-bearing precursors promote hybrid N-A-S-H/C-(A)-S-H assemblages that modify reaction kinetics and microstructural stability. Durability performance in WGP-based binders is therefore governed largely by gel continuity, pore structure refinement, and phase stability. Moderate incorporation of finely ground WGP can improve matrix densification and reduce transport connectivity, whereas excessive replacement levels or poorly balanced mix designs may increase porosity and undermine durability. Alkali–silica reaction behaviour in these systems differs from that observed in Portland cement matrices; finely ground WGP generally participates in binder formation and often mitigates expansion, although expansion behaviour remains system-dependent and influenced by alkali dosage, aluminium availability, calcium content, and curing conditions.

Despite promising laboratory-scale results, uncertainties remain regarding long-term ageing behaviour, coupled degradation mechanisms, and field-scale durability, particularly because many studies rely on paste or mortar specimens and relatively short exposure periods. Future progress toward reliable structural application will therefore require mechanism-oriented research strategies that integrate reaction chemistry, pore structure evolution, transport behaviour, and long-term durability assessment within the R–T–A framework under realistic service conditions. While WGP offers clear environmental benefits through waste valorization and reduced reliance on virgin raw materials, these sustainability advantages must be supported by demonstrable durability and service life. By synthesising reaction mechanisms, transport processes, and ageing behaviour within a unified interpretative framework, this review provides a durability-oriented perspective for understanding the system-dependent performance of WGP-based alkali-activated binders and highlights the importance of mechanistically informed mix design for future research and practical implementation.

## Figures and Tables

**Table 1:** Classification of alkali-activated binder systems based on calcium content, dominant gel chemistry, and associated durability behaviour [1,31,32,33,34].

Binder Class	Dominant Reaction Gel	Calcium Content	Typical Precursors	Microstructural Characteristics	Dominant Durability Concerns
**Low-Calcium Geopolymers**	N-A-S-H (sodium aluminosilicate hydrate)	Typically, <5–10 wt.% CaO	Fly ash, metakaolin, calcined clays, waste glass powder (blended)	Highly cross-linked aluminosilicate framework with limited Ca participation	Drying shrinkage, efflorescence, alkali mobility
**Hybrid Alkali-Activated Binders**	N-A-S-H + C-(A)-S-H (coexisting gels)	Moderate Ca content (~10–25 wt.% CaO)	Fly ash–slag blends, WGP–GGBS blends, Ca-rich industrial by-products	Mixed gel networks; refined pore structure; variable phase stability	Shrinkage–densification trade-offs, alkali redistribution, phase decalcification
**High-Calcium Alkali-Activated Materials**	C-(A)-S-H (calcium aluminosilicate hydrate)	Typically, >25 wt.% CaO	Ground granulated blast furnace slag, steel slag, lime-rich industrial residues	Hydration-dominated binding with lower polymerisation than N-A-S-H systems	Carbonation susceptibility, sulphate attack, acid-driven decalcification

**Note:** Calcium ranges are indicative and vary with precursor composition, activator chemistry, and curing conditions.

**Table 2:** Country-level glass packaging recycling performance, highlighting regional variability in collection rates and recycling quantities.

Country	Collection Rate (%)	Packaging Placed on Market (Tonnes)	Packaging Collected for Recycling (Tonnes)	Citation
USA	25.0	12,250,000	3,060,000	[37]
United Kingdom	79.0	2,416,776	1,917,818	[38]
Turkey	21.0	874,409	183,546	[39]
Switzerland	100	294,737	295,753	[38]
Sweden	85.6	254,390	217,823	[38]
Spain	75.9	1,501,346	1,140,158	[38]
Slovenia	80.2	47,552	38,117	[38]
Slovakia	79.4	104,721	83,186	[38]
Singapore	7.6	79,000	6000	[40]
Romania	67.7	442,739	299,799	[38]
Portugal	56.9	441,415	251,277	[38]
Poland	69.7	1,338,487	932,847	[38]
Norway	96.0	93,007	89,721	[38]
Netherlands	80.6	499,000	402,000	[38]
Malta	43.3	17,967	7779	[38]
Luxembourg	91.5	29,320	26,819	[38]
Lithuania	69.6	84,490	58,772	[38]
Latvia	75.4	39,975	30,131	[38]
Italy	90.8	2,642,000	2,400,000	[38]
Ireland	83.6	169,105	141,300	[38]
India	45.0	21,000,000	9,450,000	[41,42]
Hungary	29.8	175,577	52,333	[38]
Greece	34.6	107,000	37,000	[38]
Germany	85.3	2,910,700	2,481,800	[38]
France	83.7	2,745,875	2,298,297	[38]
Finland	99.2	49,110	48,738	[38]
Estonia	76.6	39,427	30,185	[38]
Denmark	86.5	207,022	179,081	[38]
Czech Republic	81.0	223,053	180,637	[38]
Cyprus	49.0	20,772	10,174	[38]
Croatia	62.5	81,248	50,742	[38]
China	44.2	22,750,000	10,050,000	[43]
Bulgaria	72.8	103,875	75,635	[38]
Belgium	92.0	363,731	334,753	[38]
Austria	87.0	290,500	252,700	[38]
Australia	80.1	1,063,383	851,406	[44]

**Note:** Minor discrepancies between “placed” and “collected” may reflect reporting year differences.

**Table 3:** Physical characteristics of waste glass powders reported in cementitious and alkali-activated systems.

Citation	Waste Glass Type	Binder System/Application	Specific Gravity (–)	True Density (g/cm³)	Particle Size Descriptor	Particle Size (µm)	Surface Area Descriptor	Surface Area (m²/kg)	Characterization Method
[9]	Green soda-lime glass powder	SCM in cement paste	NR	2.50	Size class	63–75	Blaine fineness	53	Sieving; Blaine apparatus
		SCM in cement paste	NR	2.50	Size class	25–38	Blaine fineness	126	Sieving; Blaine apparatus
		SCM in cement paste	NR	2.50	Size class	0–25	Blaine fineness	476	Sieving; Blaine apparatus
[13]	Recycled container glass powder	Ultra-high-performance concrete (UHPC)	NR	2.53	Mean particle size	25.80	Blaine fineness	335	Laser diffraction; Blaine apparatus
[17]	Fluorescent lamp glass powder	Fly ash geopolymer paste	2.55	NR	D50	4.65	Blaine fineness	1003	PSD analysis; Blaine apparatus
	Container glass powder	Fly ash geopolymer paste	2.53	NR	D50	11.72	Blaine fineness	589	PSD analysis; Blaine apparatus
[27]	Soda-lime glass powder	Glass-based geopolymer binder	2.40	NR	Mean particle size	17	Blaine fineness	592	Blaine apparatus
[46]	Recycled glass powder (RWGP16)	Cement-based materials	NR	2.46	D50	15.91	Blaine fineness	391	Helium pycnometer; laser diffraction; Blaine apparatus
	Recycled glass powder (RWGP18)	Cement-based materials	NR	2.46	D50	18.34	Blaine fineness	333	Helium pycnometer; laser diffraction; Blaine apparatus
	Recycled glass powder (RWGP25)	Cement-based materials	NR	2.46	D50	25.41	Blaine fineness	259	Helium pycnometer; laser diffraction; Blaine apparatus
[47]	Green glass powder	Cement paste	NR	2.50	D50	20.40	Blaine fineness	317	Helium pycnometer; laser diffraction; Blaine apparatus
	Brown glass powder	Cement paste	NR	2.52	D50	21.18	Blaine fineness	316	Helium pycnometer; laser diffraction; Blaine apparatus
	Transparent glass powder	Cement paste	NR	2.51	D50	28.76	Blaine fineness	299	Helium pycnometer; laser diffraction; Blaine apparatus
	Mixed-colour glass powder	Cement paste	NR	2.51	D50	22.52	Blaine fineness	312	Helium pycnometer; laser diffraction; Blaine apparatus
[48]	Silica-rich glass powder	SCM in cement mortar	NR	2.97	D50	21.92	Blaine fineness	428	Laser particle analyser; Blaine apparatus
[49]	Industrial milled waste glass powder (GP)	White high-performance concrete	NR	2.53	D50	34.78	Blaine fineness	168	Volumeter; laser diffraction; Blaine
	Further-milled glass powder (GPf)	White high-performance concrete	NR	2.50	D50	8.24	Blaine fineness	660	Volumeter; laser diffraction; Blaine

**Note:** NR indicates that the property was not reported in the original study. Particle size descriptors vary across studies. Some authors report mean particle size, while others report median particle size (D50) obtained from particle size distribution (PSD) analysis, or size-class fractions obtained through sieving. These descriptors are retained as reported to preserve the original experimental characterisation. Surface area values correspond to Blaine fineness unless otherwise specified by the authors. True density measurements were obtained using different techniques, including volumetric methods and a helium pycnometer, depending on the study. Multiple rows for the same reference represent different particle size fractions or grinding conditions investigated within the same study. The true density of most soda-lime waste glass powders reported in the literature typically ranges between 2.46 and 2.53 g/cm^3^, although deviations may occur depending on glass composition and measurement method.

**Table 4:** Reported chemical composition (oxide wt.%) of waste glass powder used in geopolymer and alkali-activated binder systems.

Citations	Material Chemical Properties										
	SiO_2_	Al_2_O_3_	Fe_2_O_3_	CaO	MgO	Na_2_O	K_2_O	SO_3_	TiO_2_	P_2_O_5_	LOI
[24]	68.23	1.11	1.18	8.26	4.06	14.57	0.21	0.19	0.05	<0.01	2.04
[22]	69.77	1.42	0.29	10	0.67	12.41	0.12	0.19	-	-	5.13
[25]	69.48	1.03	0.57	7.67	4.45	-	0.27	-	-	-	0.83
[28]	83.34	-	-	7.28	-	9.38	-	-	-	-	-
[16]	85.44	2.4	0.35	10.52	0.14	-	-	-	-	-	0.21
[30]	71.58	0.81	0.11	8.41	3.94	13.8	0.47	-	0.04	-	-
[23]	70.52	2.22	0.51	10.82	1.41	11.93	0.28	0.10	-	-	-
[17]	70.30	1.90	0.42	12.30	1.68	12.80	0.23	0.07	-	-	0.68
[27]	69.60	2.20	0.90	11.60	0.40	12.03	0.40	-	-	-	-
[18]	68.33	1.93	0.36	11.90	1.30	14.65	0.70	0.09	0.062	-	1.34
[13]	72.76	1.67	0.79	9.74	2.09	12.56	0.76	0.10	0.04	0.02	1.00
[20]	68.73	1.90	0.40	11.99	1.30	14.69	0.70	0.10	0.10	-	1.36
[50]	77.42	0.86	0.36	10.46	1.34	7.54	0.40	0.34	-	-	1.28
[51]	69.7	0.64	0.3	8.01	2.89	19.95	0.17	0.63	-	-	-
[52]	70.30	2.20	0.82	10.64	0.96	11.21	-	0.33	-	-	-

**Table 5:** Summary of durability effects of waste glass powder in geopolymer and alkali-activated binders.

Durability Domain	Typical Tests/Indicators	Low-Ca Systems (FA/MK Dominated; CaO Typically <~5 wt.%)	Ca-Rich Blends (Slag-Containing Systems; Typically, ~30–60% GGBS)	Mechanistic Drivers	Key WGP-Sensitive Parameters	Citations
Sulphate resistance	Mass change; residual strength after Na_2_SO_4_/MgSO_4_ exposure; cracking/visual rating	Sulphate resistance is commonly reported to remain stable or improve when **~10–20% WGP** replaces conventional precursors, primarily due to pore refinement and additional silica contributing to N-A-S-H gel formation. At higher replacement levels (**>~30%**), reduced availability of reactive aluminosilicates may limit gel development and compromise durability.	In hybrid systems containing **~30–60% slag**, incorporation of **~10–20% WGP** may enhance sulphate resistance through matrix densification; however, high Ca contents can lead to instability of Ca-bearing phases during prolonged sulphate exposure.	Reduced permeability through micro-filler packing and silica dissolution; durability governed by gel chemistry and pore connectivity.	WGP fineness (<75–100 µm typical); replacement level (~10–30%); slag fraction; curing regime	[3,4,5,14,27,46,57]
Acid resistance	Mass loss; strength loss in H_2_SO_4_/HCl; surface degradation	Low-Ca geopolymer matrices incorporating **~10–30% WGP** generally exhibit favourable resistance to acidic environments because N-A-S-H networks contain minimal calcium and are less susceptible to decalcification.	In Ca-rich systems containing significant slag (~30–60%), additions of **~10–20% WGP** may maintain moderate acid resistance, although strong acids can progressively degrade C-A-S-H or hybrid gels through decalcification.	Acid durability correlates strongly with Ca content and gel stability; aluminosilicate frameworks generally show greater acid tolerance than Ca-rich hydrates.	Ca fraction (slag %); WGP replacement (~10–30%); exposure pH and acid type; curing	[4,5,32,33,56,57]
Water absorption/sorptivity	Water absorption (%); sorptivity coefficient; capillary uptake	Water absorption and sorptivity often decrease when **~10–20% fine WGP (<75–100 µm)** is incorporated, due to improved particle packing and enhanced gel formation. Replacement levels exceeding **~30–40%** may increase porosity because of dilution of reactive aluminosilicate phases.	Similar reductions in absorption are reported in hybrid slag systems (~30–60%) when **~10–20% WGP** is added; excessive replacement may reduce reactive Ca/Al availability and increase capillary porosity.	Micro-filler packing combined with dissolution of glass-derived silica enhances matrix densification; excessive WGP may limit reaction extent.	WGP fineness; replacement level (~10–30%); activator modulus; curing regime	[3,4,5,22,46,48,49]
Chloride transport resistance	Chloride diffusion/migration; electrical resistivity; permeability proxies	Limited but increasing evidence suggests chloride diffusion may decrease when **~10–20% WGP** refines pore structure and increases tortuosity.	In FA–slag or slag-rich binders (~30–60% slag), incorporation of **~10–20% WGP** has been reported to reduce chloride diffusion coefficients through matrix densification and reduced connectivity of capillary pores.	Chloride transport is controlled by pore connectivity and tortuosity; hybrid gel formation reduces connected porosity when the reaction proceeds effectively.	WGP fineness; slag fraction; alkali dosage; curing regime; water/binder ratio equivalents	[3,14,20,46,58]
Efflorescence/alkali leaching (serviceability risk)	Visual efflorescence rating; leachate alkalinity; Na/K loss	Efflorescence risk may increase when WGP content is high (**>~30%**) or when alkali activation is excessive, and reaction remains incomplete, leaving free alkalis in the pore solution. Moderate WGP levels (**~10–20%**) may reduce transport pathways through matrix densification.	Similar behaviour is observed in Ca-containing systems; denser hybrid matrices may reduce alkali transport, although moisture gradients can still drive surface salt formation.	Controlled by free alkali availability, pore solution chemistry and moisture transport, immobilisation improves when alkalis are incorporated into N-A-S-H or hybrid C(N)-A-S-H gels.	Activator concentration; WGP replacement; curing humidity; Si/Al ratio	[4,5,33,53,59]
ASR-type expansion risk (WGP as powder, not aggregate)	Expansion measurements; cracking; petrography; gel identification; pore solution analysis	Fine WGP particles (**<75–100 µm**) typically dissolve and participate in geopolymer gel formation rather than acting as reactive aggregate; expansion risk is therefore generally lower when sufficient Aluminium is available.	Behaviour can vary depending on alkali concentration, Aluminium availability and Ca content. Some Ca-rich systems report low expansion, but expansion behaviour remains system-dependent.	AAM pore solution chemistry differs fundamentally from OPC systems; WGP generally acts as a reactive silica precursor rather than an aggregate.	Particle size; alkali dosage; Aluminium availability; curing regime	[4,5,45,60,61,62]
Shrinkage/dimensional stability	Drying shrinkage strain; autogenous shrinkage; cracking	Silica-rich matrices incorporating **~10–30% WGP** may show increased autogenous or drying shrinkage due to refined pore networks and higher capillary stresses.	In slag-rich systems (~30–60%), WGP additions (**~10–20%**) may either reduce drying shrinkage through filler effects or increase autogenous shrinkage when densification becomes significant.	Shrinkage governed by pore size distribution, reaction extent and curing conditions; densification reduces permeability but may increase capillary stress.	Activator modulus; WGP content; slag fraction; curing humidity	[4,5,14,46,58]
Long-term ageing/coupled degradation	Long-term strength/transport evolution; wet-dry cycling; multi-ion exposure	Long-term field data remain limited. Some studies suggest partial dissolution of residual WGP particles may provide additional silica that contributes to gradual gel evolution under favourable curing conditions.	Similar behaviour may occur in hybrid systems; however, Ca-phase alteration or leaching may occur under aggressive exposure environments.	Ageing is governed by gel reorganisation, leaching and transport processes; durability outcomes remain exposure dependent.	Exposure duration; cyclic environmental conditions; specimen scale; WGP replacement level	[4,5,29,32,33,51,57]

## Data Availability

No new data were created or analysed in this study. Data sharing is not applicable to this article.

## Abbreviations

The following abbreviations are used in this manuscript:

AAM	Alkali-Activated Material
ASR	Alkali–Silica Reaction
C-S-H	Calcium Silicate Hydrate
C-(A)-S-H	Calcium (Alumino)Silicate Hydrate
C(N)-A-S-H	Calcium (Sodium) Aluminosilicate Hydrate
DTG	Derivative Thermogravimetry
FA	Fly Ash
FTIR	Fourier Transform Infrared Spectroscopy
GGBS	Ground Granulated Blast Furnace Slag
LOI	Loss on Ignition
MK	Metakaolin
N-A-S-H	Sodium Aluminosilicate Hydrate
OPC	Ordinary Portland Cement
R–T–A	Reaction–Transport–Ageing
SCM	Supplementary Cementitious Material
TG	Thermogravimetric Analysis
WGP	Waste Glass Powder
